# In Vitro and In Vivo Evaluation of a Nano-Tool Appended Oil_mix_ (Clove and Tea Tree Oil) Thermosensitive Gel for Vaginal Candidiasis

**DOI:** 10.3390/jfb13040203

**Published:** 2022-10-26

**Authors:** Abdulrab Ahmed M. Alkhanjaf, Md Tanwir Athar, Zabih Ullah, Ahmad Umar, Ibrahim Ahmed Shaikh

**Affiliations:** 1 Department of Clinical Laboratory Sciences, College of Applied Medical Sciences, Najran University, Najran 11001, Saudi Arabia; 2 College of Dentistry and Pharmacy, Buraydah Colleges, Buraydah 51452, Saudi Arabia; 3 Department of Chemistry, Faculty of Science and Arts, and Promising Centre for Sensors and Electronic Devices (PCSED), Najran University, Najran 11001, Saudi Arabia; 4 Department of Materials Science and Engineering, The Ohio State University, Columbus, OH 43210, USA; 5 Department of Pharmacology, College of Pharmacy, Najran University, Najran 64462, Saudi Arabia

**Keywords:** nanoemulsion, pseudo-ternary phase diagram, ex vivo permeation study, MTT assay, mucoadhesion

## Abstract

The main objective of the proposed work was the development of a thermosensitive gel (containing clove and tea tree oil) for the management of vaginal candidiasis. Both oils have been recommended to be used separately in a topical formulation for vaginal candidiasis. Incorporating two natural ingredients (clove and tea tree oil) into a product give it a broad antimicrobial spectrum and analgesic properties. The two oils were mixed together at a 3:1 ratio and converted into o/w nanoemulsion using the aqueous titration method and plotting pseudo ternary phase diagrams. Further transformations resulted in a gel with thermosensitive properties. To determine the final formulation’s potential for further clinical investigation, in vitro analyses (viscosity measurement, MTT assay, mucoadhesion, ex vivo permeation) and in vivo studies (fungal clearance kinetics in an animal model) were conducted. The current effort leveraged the potential of tea tree and clove oils as formulation ingredients and natural therapeutic agents for vaginal infections. Its synergy generated a stable and effective thermosensitive gel that can be utilized for recurrent candidiasis and other infections.

## 1. Introduction

Vaginal candidiasis is the second most common vaginal infection, and up to 30% of pregnant women are estimated to suffer from vaginal candidiasis [1]. As per an estimation, every other woman will have had at least one urinary tract infection during her lifetime, the risk of which increases with age [2]. The most common symptoms are itching, dyspareunia, soreness, pruritus, vulvar erythema and oedema. Host factors, such as hormonal changes, the prolonged use of antibiotics and disturbances in the vaginal ecosystem, appear to be the main causative factors of the condition [3].

The commonly recommended treatment is through the oral route using azole antifungals. Chances of recurrent infection are always present, and recurrence may be attributed to bacterial or fungal biofilms [4]. So, topical application is also required for a two-pronged treatment approach, i.e., oral and topical. The use of herbal actives may always be advantageous if not irritable. Now herbal products are also being used for the same approaches [1,5,6]. Some utilized herbs reported in the literature are thyme, garlic, olive oil, propolis, myrtus, black cumin and black seeds. Tea tree oil (TTO) and clove oil (CO), both separately and in combination, have been recommended for antifungal preparations [7]. However, the combination could be an irritant to the soft tissues, such as the vaginal tissue [8,9]. An exhaustive literature search revealed that either the oils or their ingredients have already been explored for vaginal applications [10,11,12]. So, a concept was hypothesized to develop a non-irritable combination and then convert it into a patient-complaint gel. The amount was kept to a minimum and encapsulated into the globules of o/w nanoemulsion, which was further converted into a thermo-sensitive gel (i.e., it remains liquid at room temperature but converts into gel at body temperature). The small size range (i.e., nanoscale) helps with the penetration into mucus layer [13] and dealing with deep-seated fungal infections. In addition to this, the incorporation of actives, such as TTO and CO, into the lipid phase confers drug-protective characteristics against the biocatalytic constituents of mucus and fluids [14]. Other researchers have also suggested the same strategy to deal with the irritating effect of oils [15]. Gels are supposed to be a better-tolerated delivery system compared to other dosage forms, such as inserts or ointments [16], and the imparting of the thermosensitive property was an added advantage.

The given work was designed to prepare a nanoemulsion (NE) of a mixture of oils, i.e., TTO and CO, at a ratio of 3:1. The intermediate product NE (also known as submicron emulsions, ultrafine emulsions, mini-emulsions) may range from 1 nm–1000 nm (by the definition of USFDA and EMA), but in practicality, a size range of 20–200 nm with a narrow size distribution was considered for product development [17]. For the given project, it was o/w emulsion type, NE is considered a suitable carrier owing to its properties, such as evading the enzymatic attack, protection against oxidation and hydrolysis, and minimizing the irritation potential of the encapsulated actives [18,19]. After successful development, the NE (intermediate product) was converted into a thermosensitive gel that responds to the temperature, i.e., remains liquid at room temperature but converts into gel texture at body temperature [20]. Thermosensitive gels for drug delivery have been reported in the literature with different active ingredients [21,22]. The advantage of using a gelling system is that it remains localized for a sufficient period and controls and prolongs drug release. Imparting bioadhesive properties to a gel further improves it. Gels that are used as contraceptives, microbicides, labour-inducers, and pH-restorers during menopause are receiving a significant amount of attention from researchers working on vaginal drug delivery systems, as is evident from the existing body of research [23,24]. 

## 2. Materials and Methods

### 2.1. Materials 

CO and TTO were purchased from a reputable local supplier. Gattefosse Pharmaceuticals (India) provided Labrasol as a free sample. Sigma-Aldrich provided the Poloxomer 407. Carbopol 940 and Tween 20 were procured from SD Fine Chemicals (Mumbai, India). All excipients and chemicals used were of analytical grade.

### 2.2. Cytotoxic Potential of Oil_mix_

The selected oils (CO and TTO) are irritating to soft tissues. Therefore, MTT assay was performed as per a previously reported method [22]. In brief, the cytotoxicity of Oil_mix_ (3:1 *w*/*w* ratio) was evaluated by MTT assay, using HeLa-S3 cell lines. The cell viability was calculated using the formula:

$$\mathrm{Cell}\;\mathrm{viability}\;\left(\%\right)=\frac{\mathrm{Optical}\;\mathrm{Density}\;\mathrm{of}\;\mathrm{test}\;\mathrm{sample}}{\mathrm{Optical}\;\mathrm{Density}\;\mathrm{of}\;\mathrm{control}\;\mathrm{sample}}\times 100$$

### 2.3. Preparation of Nanoemulsion Formulations

#### 2.3.1. Construction of Pseudo Ternary Phase Diagram 

The NE served as an intermediate which was further converted into the finished product, i.e., a thermo-sensitive gel. Tween 20 was chosen as a surfactant. Labrasol was taken as a co-surfactant. Oil_mix_, S_mix_ (surfactant-co-surfactant mixture), and distilled water were used in the pseudo ternary phase diagrams. The aqueous titration method was used to create it. The surfactant and co-surfactant were mixed in various weight ratios (1:0, 1:2, 1:1, 2:1, 3:1, 1:3, and 4:1), which were chosen based on the increasing concentration of surfactant relative to co-surfactant and the increasing concentration of co-surfactant relative to surfactant. In separate glass vials, oil and a specific S_mix_ ratio were mixed properly in different weight ratios ranging from 1:9 to 9:1. Sixteen different combinations of oil and S_mix_ (1:9, 1:8, 1:7, 1:6, 1:5, 1:4, 1:3.5, 1:3, 1:2.3, 1:2, 1:1.5, 1:1, 1:0.7, 1:0.43, 1:0.25, and 1:0.1) were used to define the boundaries of phases specifically formed in each phase diagram. Different o/w NE were formed by slowly titrating Oil_mix_ and S_mix_ in an aqueous phase and evaluating their transparency and flowability. The phase diagrams were used to represent the physical state of the nanoemulsion, with one corner representing the aqueous phase, the other representing Oil_mix_, and the third representing S_mix_. In each phase diagram, the nanoemulsion area was plotted, and the larger the region, the better the nano-emulsifying efficiency. 

#### 2.3.2. Phase Diagram-Based Selection of Formulations

##### Thermodynamic Stability Test

Thermodynamic stability tests were performed to address the issue of metastable formulation [25]. To check for phase separation, creaming, or cracking, a few different formulations were centrifuged (REMI, India) at 5000 rpm for 30 min. Only the formulations that did not undergo phase separation during the heating and cooling cycle were used. Each temperature was maintained for no less than 48 h over the course of six cycles: refrigerator temperature 0 °C and 45 °C. These freeze–thaw cycles were applied to formulations that were stable in this temperature range. Selected formulations were subjected to three freeze–thaw cycles between −21 °C and +25 °C, with storage at each temperature for no less than 48 h. 

### 2.4. Characterization of Selected Nanoemulsion Formulations

Selected formulations were characterized for the following in vitro attributes.

#### 2.4.1. Globule Size Analysis

Using a laser light scattering phenomenon-based photon correlation spectrometer (Zetasizer 1000 HAS, Malvern Instruments, Worcestershire, UK), the globule size of the nanoemulsion was calculated by analysing the fluctuations in light scattering. At 25 °C and a 90-degree angle, light scattering was measured. Nanoemulsion samples were properly diluted (0.1 mL) and dispersed (in 50 mL) in water for droplet size analysis. The typical size of droplets and their polydispersity index were measured. A total of three measurements were taken. The samples were also kept undisturbed for two weeks to check for any increase in size or size ripening. 

#### 2.4.2. Viscosity

The rheological properties of various formulations were measured at 25 ± 1.0 °C using a Brookfield DV III ultra V6.0 RV cone and plate rheometer (Brookfield Engineering La-boratories Inc., Middleboro, MA, USA, spindle # CPE40). 

#### 2.4.3. Surface Morphology

A 200 kV transmission electron microscope (TEM) (TOPCON 002B) with a subnanometre resolution was used to examine the surface morphology of nanoemulsion. For TEM analysis, we used nanoemulsion system samples that were appropriately diluted (1/100 in water). After the diluted nanoemulsion was dropped onto the copper film grid, it dried into a circular pattern. The shape and size of the nanoemulsion were determined using a mix of bright field imaging at increasing magnification and diffraction modes.

#### 2.4.4. Refractive Index

The refractive index of selected formulations was determined using an Abbe-type refractometer. Standardization was performed using castor oil.

##### pH Measurement

The pH of the nanoemulsion that gives an idea about the potential irritation was measured by a pH meter (Mettler Toledo MP 220, Greifensee, Switzerland) in triplicate at 25 °C. 

### 2.5. Development of Thermosensitive Gel

It is reported that patients better tolerate gels than inserts or ointments in the case of vaginal application [16]. Hence the optimized nanoemulsion was converted into a gel, using Carbopol 934 (CP 934) and Poloxamer 407 (P 407). Carbopols are well accepted in vaginal drug-delivery and exhibit mucoadhesion as well. P 407 offers thermo-sensitive properties, i.e., it remains in solution form at room temperature but attains gel texture at body temperature and at a particular concentration. Furthermore, P 407 has low toxicity potential and good compatibility with other excipients. In brief, Carbopol 934 was slowly added to citrate phosphate buffer (0.1 M) having pH 4.0 at 4 °C with gentle mixing for 4 h and allowed to hydrate. The air bubbles were removed by centrifuging at 4000 rpm for 15 min. It was also taken care of during mixing to have negligible air bubbles. P 407 (at concentrations of 16%, 18%, and 20%, *w*/*v*) was then added to the solution and allowed to dissolve overnight at 4 °C. A calculated amount of NE was then added, mixed, and stored in a refrigerator. The amount of Oil_mix_ (incorporated in NE) in the finished product was kept constant (i.e., 1%, *w*/*v*). The total amount of CP 934 was kept constant (0.4%, *w*/*v*) in all three batches (Table 1). Conventional gels were prepared separately for TTO (0.75%, *w*/*v*) and CO (0.25%, *w*/*v*), using the same S_mix_ to disperse the oils. 

#### 2.5.1. Characterization and Evaluation of Optimized Finished Formulation (Thermo-Sensitive Gel)

As optimization tools, different gel compositions were evaluated for the following in vitro parameters. 

#### 2.5.2. Sol-Gel Transition Temperature

To measure the sol-gel transition temperature, 5 mL of the liquid composition and a small magnetic bead were placed in a 20 mL glass vial, which was then kept in a water bath. It was heated at a rate of 2 °C/min with continuous stirring of 150 rpm. The temperature point at which the bead stops rotating is the gelation temperature. 

#### 2.5.3. Viscosity Measurement

The viscosity measurement was carried out according to the method described previously. The nature of flow under the shear was determined.

#### 2.5.4. Mucoadhesion Test Using Texture Analyzer

The gel formulation underwent a mucoadhesion test, as described in the literature [22]. The formulation was tested for its maximum detachment force (MDF) from a cellophane membrane that had been pretreated with simulated vaginal fluid (SVF). The TA-XT2 Texture Analyzer r (Stable Micro Systems, Godalming, UK) is a highly accurate, computer-operated system for analysing surface textures. Gels were prepared in batches and sandwiched between cellophane membranes saturated with SVF and attached horizontally to the bottom and top probes. The experiment was repeated three times at a temperature of about 37 ± 1 °C. When the two contact phases (the cellophane membrane and the mucoadhesive gels) formed an interface of unit area and were subsequently separated reversibly and resulted in an increase in surface area, the work performed on the matrices was characterized by the adhesion work per unit area. Maximum detachment force (MDF) is the force required to detach a polymer or dosage form from a substrate after contact at a given time and force, and the instrument measures this to determine the work of adhesion. The system displays the force of detachment, referred to as the maximum detachment force (MDF). 

### 2.6. Ex Vivo Drug Permeation Study

Franz diffusion method was used to carry out ex vivo permeation [26]. An amount of 1 mL of different compositions of the batches (B2, B3, and B4) was placed into the donor compartment with 0.75 mL of simulated vaginal fluid. It was mimicking the vaginal milieu. An amount of 20 mL phosphate of buffer (pH 4.5) was added into the receptor compartment. At regular intervals samples were withdrawn (0.5, 1, 2, 4, 6, 8, 10, 12, 14, 16, and 24 h). The withdrawn samples were filtered through a 0.45 µm membrane filter and analysed by HPTLC. The authors did not find a simultaneous method, so they separately analysed both the oils/markers. For the analysis of clove oil, the previously described method was followed [27], and for the analysis of TTO, Biju et al., 2005, was followed [28]. The development of a simultaneous method was outside of the scope of work. The reported analytical methods were validated before application. The cumulative amount of oils (TTO and clove oil) permeated (g/cm^2^) was plotted as a function of time (t) for each gel, and the permeability parameters were determined (Table 2).

### 2.7. In Vivo Antifungal Studies of Optimized NE Gel

In vivo testing was performed using a rat model, a method that has been well-documented [22]. The Scientific Ethical Committee at Najran University gave their permission for the research (Project code 443-42-59216-DS). All experiments were conducted in compliance with international guidelines for the humane treatment of animals (National Institutes of Health Publications No. 8023, revised in 1978). The female rats (n = 24) were divided into four groups. Table 3 provides a breakdown of the groupings.

The rats were kept in polypropylene cages and provided with a standard diet (Lipton feed, Mumbai, India) and water ad libitum in a controlled laboratory environment. All animals were kept in pseudo-oestrus with subcutaneous oestradiol benzoate injections for six days prior to the inoculation of the infection. Oophorectomized rats were inoculated with a saline solution containing 108 colony forming units (CFU) of *Candida albicans* per millilitre. One gram of each gel was applied a day later. Using a syringe, 1 µL of vaginal fluid was collected from each animal at regular intervals, diluted, and spread on Sabouraud agar with chloramphenicol (50 mg/mL). At 32 degrees Celsius, the inoculum was kept in a BOD incubator. When the number of new infections was low enough, the researchers initiated their experiment. A rat is said to be infected when there is at least one CFU per millilitre of fluid (i.e., a count of ≥103 CFU/mL) in the sample. Vaginal fluid CFU counts were plotted against time or day for a visual representation of the data and a basis for comparison. Data were analysed using GraphPad Prism version 6 (San Diego, CA, USA). The results are expressed as mean ± SD. The differences between various groups were calculated using one-way analysis of variance (ANOVA) followed by Tukey’s post hoc test. A *p*-value less than 0.05 was considered to be significant.

## 3. Results and Discussion

### 3.1. Cytotoxic Potential of Oil_mix_

The data of the cytotoxicity potential of the Oil_mix_ (3:1 *w*/*w* ratio) were acceptable as there is no major sign of toxicity. The obtained data were analysed by Graph pad prism 5. IC_50_ data obtained were 39.44 µg/mL. Data were obtained for 24 h only, as similar studies suggest that for a similar type of active ingredient, a 24 h period is sufficient for complete fungal clearance [22].

Both the oils (TTO and CO) are mentioned in the natural medicines database (https://naturalmedicines.therapeuticresearch.com/ Last accessed: 20 August 2022). The quantities of active and excipients taken in the project were well within the limits suggested by regulatory databases or approved product lists. The USFDA database recommends a maximum amount of 2.4% *w*/*w* for CO. The Medsafe database recommends TTO up to 25% for topical application. The maximum used amount of Tween 20 for vaginal application as per the USFDA database is 3% *w*/*w*, having GRAS status also included in the Canadian list of acceptable non-medicinal ingredients.

### 3.2. Preparation of Nanoemulsion Formulations

It is well accepted that for a typical o/w nanoemulsion, the value of the required HLB is more than 10. Hence, a mixture of Tween 20 and Labrasol was chosen as a surfactant and co-surfactant, respectively. Ionic surfactants were deliberately not utilized for the study because of irritation potential, higher critical micelle concentration values, and pH susceptibility. The application site (vaginal tissue) is very sensitive, so care must be taken when selecting active and inactive excipients. Furthermore, the pH does not remain constant throughout the vaginal cavity but is the highest near the cervix and the lowest near the anterior fornix.

**Figure 1:**
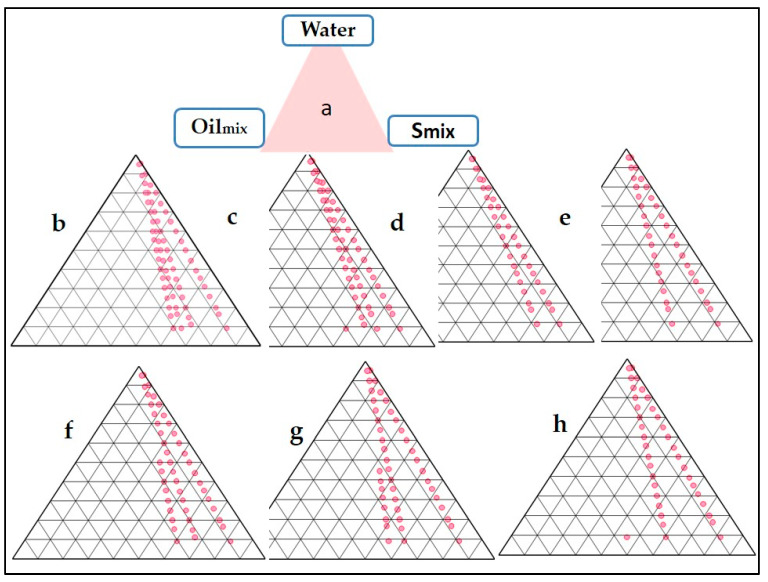
Pseudo-ternary phase diagram depicting Oil_mix_, Tween 20 (surfactant), and Labrasol (co-surfactant) at varying S_mix_ ratios (Group (a) to Group (c) on a pseudo-ternary phase diagram based on aqueous titration (h).

The areas of phase diagrams give an idea about the effect of a particular composition of S_mix_ over the NE formulation (Figure 1). Larger areas indicate a more significant number of nanoemulsion formations. Ideally, the NE should be spontaneous and stable; hence, metastable formulations are not selected. There was no fixed pattern of the NE area when the ratio of either the surfactant or co-surfactant was increased. The maximum area was obtained at a S_mix_ ratio of 2:1 and 3:1. It can be concluded that the NE formulation, in this case, is independent of surfactant composition. The negative free energy of formation is achieved when significant favourable entropic changes accompany a large reduction in surface tension. In such a case, the NE formation is spontaneous, and the resulting dispersion is thermodynamically stable [22]. Despite metastability, nanoemulsions can persist over many months or years due to a stabilizing surfactant inhibiting the nano-droplet coalescence [29]. The reduction of droplet sizes to the nanoscale leads to some interesting properties, such as optical transparency and unusual elastic behaviour. The surfactant and co-surfactant mass ratio was a key factor influencing the phase properties, i.e., the size and position of the nanoemulsion region [30].

#### 3.2.1. Selection of Formulations from Phase Diagrams

The thermodynamic stability test exposes the different compositions to stress conditions. A composition is supposed to pass the test if there is no phase change and it remains homogenous and transparent. Out of the different stable compositions obtained, the optimized ones were selected based on the smallest quantity of surfactant, i.e., 10% Oil_mix_, 35% S_mix_ (2:1), and 55% water. This was an intermediate product for further development into a thermosensitive gel. The usual preference is to select formulations with the lowest surfactant concentration, which can be stretched up to recommended limits. Another criterion for surfactant selection is the concentration which gives maximum flux. Since a high concentration of surfactant reduces the drug’s thermodynamic activity in the vehicle and increases the drug’s affinity to the vehicle, this effect is typically not seen with formulations containing the highest possible amount of surfactant [31].

#### 3.2.2. Characterization of Selected Nanoemulsion Formulations

**Figure 2:**
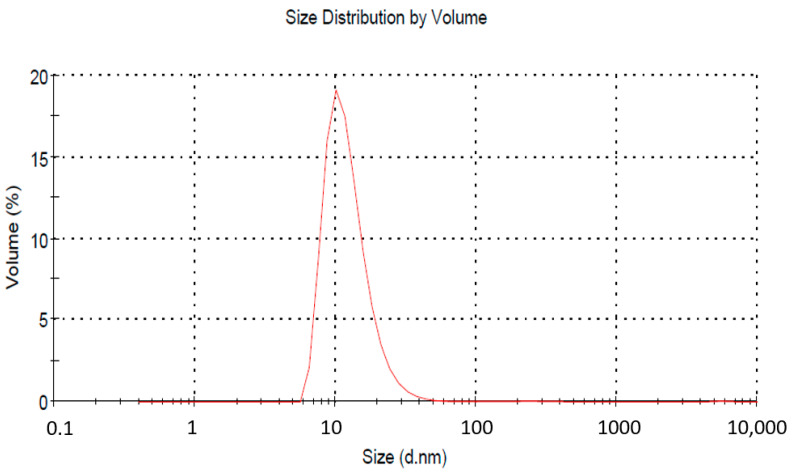
Globule size analysis of nanoemulsion by zetasizer. The average droplet size was 62.11 nm, with a PDI of 0.359.

The acceptable size range as per the regulatory definition is up to 1000 nm, but for practical purposes, a size below 200 nm is taken. The average droplet size in our case was 62.11 nm with a PDI of 0.359 (Figure 2). The zeta potential of the NE was recorded as −40.35 mV. Surfactants play a dual role in maintaining the stability of an emulsion. They offer stearic hindrance as well as repulsive surface potential between globules. The lower value of zeta potential indicates better stability. To further confirm the NE size’s stability, analysis was conducted again after two weeks. A slight increase in average particle size was obtained (64.41 nm). Literature reports mention a comparative larger globule size of NE for vaginal drug delivery (da Silva et al., 2021; dos Santos et al., 2020). So, the given size can be accepted for further finished product development.

**Figure 3:**
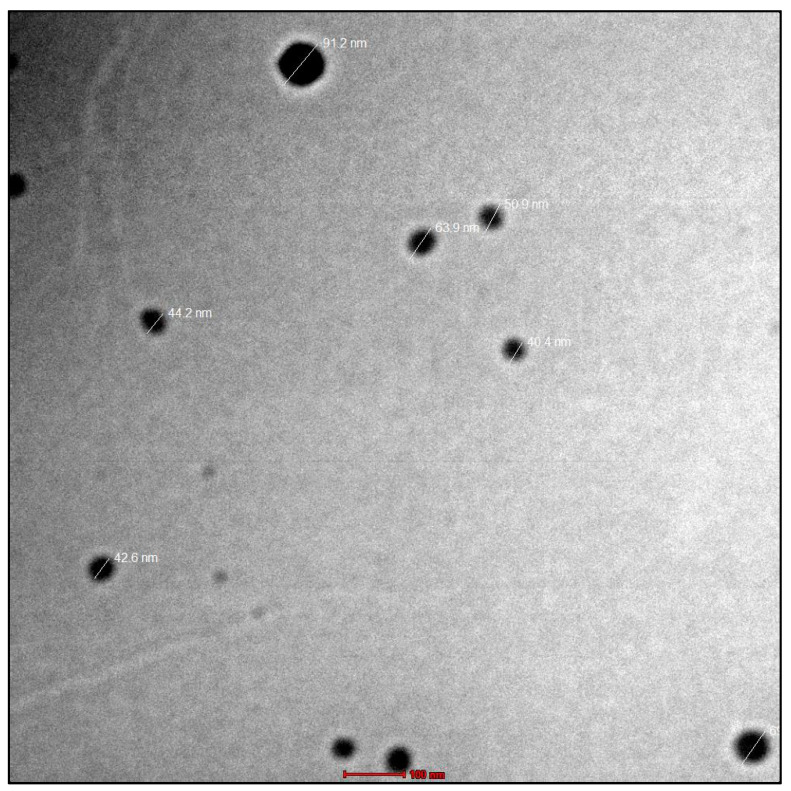
The TEM micrographs showed that the nanoemulsion droplets had spherical shape, were discrete, appeared dark, and contained an amorphous core.

The viscosity analysis showed it to be a Newtonian fluid with a viscosity of 17.23 ± 36 cps at room temperature. Furthermore, the results were confirmed using TEM imaging. Lipid emulsion droplets were found to be virtually spherical in shape, distinct and black, with an amorphous centre. Droplet diameters ranged from 40.4 to 91.2 nm, according to the measurements. The images of TEM are given in Figure 3. The refractive index was found to be 1.521 ± 0.022.

### 3.3. Evaluation of Thermosensitive Gel

#### 3.3.1. Measurement of Sol-Gel Transition Temperature and Viscosity

The gelation temperature is defined as the temperature below which the polymeric material is soluble in an aqueous medium and above which it undergoes a phase transition to increase viscosity or form a semi-solid gel. A small increase in gelation temperature was observed with every 2% increase in the amount of Poloxamer 407. The batch corresponding to human body temperature is B4, so it was taken as an optimized batch. The increase in viscosity from B2 to B4 correlates with increasing polymer content. The ideal gel platform must provide a good coating with minimal leakage. Gels are generally defined as non-Newtonian and pseudoplastic with thixotropic behaviour and yield stress.

#### 3.3.2. Mucoadhesion Test Using Texture Analyzer

The interaction between mucoadhesive materials and mucus is a result of physical bonding and other factors, such as H-bonding, anionic charges, high molecular weight, surface energy properties, chain flexibility, and van der Waals attraction. The chemical groups on the surface that contribute to mucoadhesion are generally hydroxyl, carboxyl, amine, and amide groups in the structure [32,33,34,35,36]. These factors were also found in the polymeric systems selected in our study. The result is given in Table 1. There was not much difference between the batches, but the difference in the number of polymers could be seen in terms of values of mucoadhesion. The maximum values of mucoadhesion were observed in B4.

#### 3.3.3. Ex Vivo Drug Permeation Study

The flux values of the batches were found in the range of 0.006–0.012 mg/cm^2^/h. The value of *K*_p_ is also given in the table. The values of permeation of B2 were almost double of those of B4. Since there is no need for much permeation or systemic exposure, B4 was a promising candidate. So based on the results of gelling temperature, mucoadhesion, and ex vivo permeation, B4 was selected for in vivo studies [37,38,39,40,41].

#### 3.3.4. In Vivo Antifungal Studies of Optimized NE Gel

**Figure 4:**
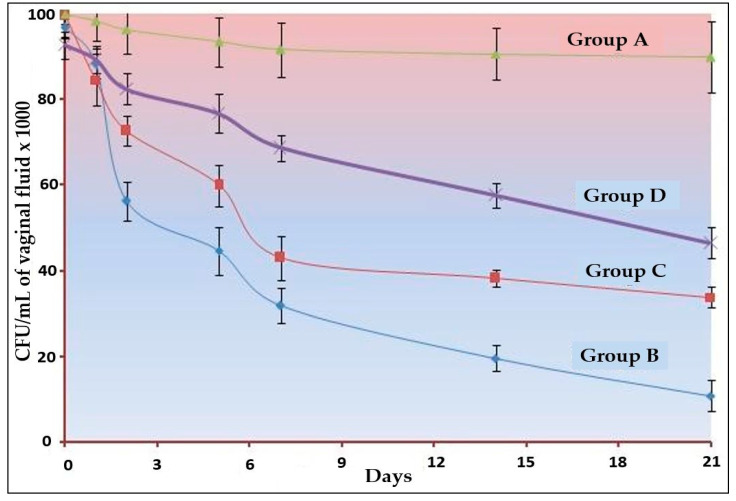
Clearance kinetics *C. albicans* infection in oestradiol-treated oophorectomized rats (n = 6). A significant difference (*p* < 0.001) was observed between all three formulations.

The results of in vivo studies are depicted in Figure 4. In the 21 days of studies, the highest clearance was observed with group B, i.e., optimized Oil_mix_ containing NE-based gel. There was a significant (*p <* 0.001) amount of clearance for the other two conventional gel (TTO and CO) but less than the Oil_mix_-based gel. There is no study incorporated for the measurement of the analgesic property of the optimized formulation, but the activity of CO can be easily observed in the form of clearance kinetics. Despite having less TTO, the clearance was higher in the Oil_mix_-based product. The reasons may be attributed to the combination effect and the nano-size range [42,43,44].

The optimized Oil_mix_ (B4) gel would most likely be less irritating for use on the skin or mucosa, but further research into skin irritation is required to substantiate this notion. The results obtained against *C. albicans* allow for the topical use of the optimized Oil_mix_ (B4) gel against candidiasis, but they should be verified using in vivo candidiasis models. However, developed NEs could potentially be employed in topical applications.

The outcomes of this study met the expectations in terms of synergism. Our synergistic optimized gel of Oil_mix_ (B4) formulation was shown to have the highest clearance, whereas the standard clove oil gel was found to have the lowest clearance. Permeability and comprehensive antibacterial action with lower MIC values may explain the greater clearance of traditional TTO gel. Almost all the clearance kinetics graph patterns were identical, which can be attributed to the drugs’ diffusion properties across the gel matrix. 

The findings from the present study are in line with the findings of previous studies (22; 14).

## 4. Conclusions

The current effort leveraged the potential of tea tree and clove oils as formulation ingredients and natural therapeutic agents for vaginal infections. Its synergy generates an effective and stable thermosensitive gel that can be utilized for recurrent candidiasis and other infections. The local application of tailored Oil_mix_ (B4) gel with proper loading may prevent a common female ailment. Positive in vitro and in vivo results may lead to clinical applications. To succeed commercially, we need to validate the results clinically with a GMP batch of the product. The selection of natural actives (TTO and CO) may have an advantage at this stage. We may not require a detailed clinical evaluation as is generally necessary with synthetic moieties. The safety parameters of the subject are also supposed to be less stringent because of its natural origins and widespread application of their components in different ailments, as well as the supporting scientific literature. The efficacy endpoints should remain the same. The given research may pave the way for evaluating further combinations of two or more natural ingredients for the given and other ailments.

## Figures and Tables

**Table 1:** Composition of different batches of gels (finished product) and their evaluation. Readings were taken in triplicate and average values have been given in the table. Batch 1 (placebo) does not contain any of the actives. The difference of composition between different batches is in amount of P 407 added.

Batch Name	Compositions (% *w*/*w*)	Gelling Temperature (°C)	Viscosity (Pa.s)	MDF (g) (Mean ± SD)	MDF (g) after Dilution (Mean ± SD)
B 1	0.4% CP 934, 16% P 407 (Placebo)	40 ± 1	0.906	-	-
B 2	0.4% CP 934, 16% P 407	40 ± 2	0.905	28.8 ± 0.4	22.5 ± 0.2
B 3	0.4% CP 934, 18% P 407	38 ± 2	0.911	30.4 ± 0.5	23.3 ± 0.6
B 4	0.4% CP 934, 20% P 407	36 ± 1	0.914	33.6 ± 0.3	25.4 ± 0.4

**Table 2:** Ex vivo permeation profile of different batches of thermo-sensitive gels. The calculation was carried out on a triplicate basis. *J*_ss_ was determined from the linear portion of the graph. *K*_p_ was obtained by dividing *J*_ss_ with the drug concentration in the donor cell (C_o_).

Batch No.	*J_ss_* ± SD (mg/cm^2^/h)	Permeability Coefficient (*K*_p_, cm/h)
B 2	0.012 ± 0.0002	0.00420021
B 3	0.007 ± 0.0019	0.00245012
B 4	0.006 ± 0.0003	0.00210011

**Table 3:** Nomenclature of groups. In all the groups (A to D) disease was induced. Different treatments were given to groups B to D, while no treatment was given to Group A during the study.

Group (n = 6)	Treatment
A	Negative control
B	Optimized gel of Oil_mix_ (B4)
C	Conventional gel of TTO
D	Conventional gel of clove oil

## Data Availability

All data has been included in the article.
